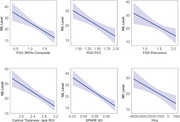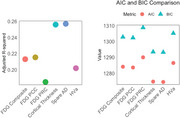# Differential relationships between plasma neurofilament light chain and various neuroimaging neurodegeneration markers

**DOI:** 10.1002/alz70856_102093

**Published:** 2025-12-24

**Authors:** Dahyun Yi, Min Soo Byun, Gijung Jung, Hyejin Ahn, Jun‐Young Lee, Yun‐Sang Lee, Yu Kyeong Kim, Koung Mi Kang, Chul‐Ho Sohn, Dong Young Lee, KBASE research group

**Affiliations:** ^1^ Institute of Human Behavioral Medicine, Medical Research Center, Seoul National University, Seoul, Korea, Republic of (South); ^2^ Seoul National University College of Medicine, Seoul, Korea, Republic of (South); ^3^ Department of Neuropsychiatry, Seoul National University Hospital, Seoul, Korea, Republic of (South); ^4^ Interdisciplinary program of cognitive science, Seoul National University, Seoul, Korea, Republic of (South); ^5^ SMG‐SNU Boramae Medical Center, Seoul, Korea, Republic of (South); ^6^ Department of Neuropsychiatry, SMG‐SNU Boramae Medical Center, Seoul, Korea, Republic of (South); ^7^ Department of Psychiatry, Seoul National University College of Medicine, Seoul, Korea, Republic of (South); ^8^ Department of Nuclear Medicine, Seoul National University College of Medicine, Seoul, Korea, Republic of (South); ^9^ Department of Radiology, Seoul National University Hospital, Seoul, Korea, Republic of (South); ^10^ Seoul National University Dementia Research Center, Seoul, Korea, Republic of (South)

## Abstract

**Background:**

Neurofilament light chain (NfL), a plasma biomarker of axonal injury, is a key indicator of neurodegeneration (N) in the A/T/N diagnostic framework for Alzheimer's disease (AD). While its clinical utility is well‐recognized, the relationship between plasma NfL and neuroimaging‐based neurodegeneration markers remains unclear. This study investigates the associations between plasma NfL and neuroimaging markers of neurodegeneration.

**Method:**

Data from 167 older adults spanning the AD diagnostic spectrum were analyzed from the Korean Brain Aging Study for the Early Diagnosis and Prediction of AD (KBASE). Plasma NfL levels were measured using the single molecule array (SIMOA) platform. Neuroimaging metrics included FDG‐PET scans (measuring hypometabolism) and structural MRI metrics such as cortical thickness, hippocampal volume, and SPARE‐AD scores. Six linear regression models assessed the associations between plasma NfL and neuroimaging markers. Model performance was evaluated using Adjusted R‐squared, Akaike Information Criterion, and Bayesian Information Criterion.

**Result:**

Plasma NfL was significantly associated with neuroimaging markers of both structural and functional neurodegeneration (Figure 1). Among MRI markers of structural neurodegeneration, reduced cortical thickness of AD signature ROIs showed the strongest association with NfL (*β* = −12.683, *p* = 3.06×10^−6^); higher SPARE‐AD scores, reflecting diffuse atrophy, also demonstrated a robust relationship (*β* = −664.953, *p* = 2.74×10^−6^). In contrast, hippocampal volume, a focal marker, showed a moderate association with NfL (*β* = −0.00315, *p* = 0.001234). Among FDG‐PET markers, lower uptake in the PCC, a region highly sensitive to early metabolic disruption in AD, showed the strongest association with NfL (*β* = −15.508, *p* = 0.000281) and the highest adjusted R‐squared (Figure 2), followed by the composite ROI, indicative of diffuse hypometabolism (*β* = −15.721, *p* = 0.000362), and the precuneus (*β* = −11.712, *p* = 0.00813), which shows metabolic decline later in AD progression. These findings show plasma NfL is more strongly associated with diffuse neurodegeneration markers, such as cortical thickness and SPARE‐AD, than focal markers like hippocampal volume or FDG precuneus. MRI markers exhibited stronger and more consistent associations with plasma NfL than FDG‐PET, though PCC hypometabolism also showed significant links.

**Conclusion:**

Plasma NfL is more strongly linked to MRI markers of diffuse structural neurodegeneration than FDG‐PET markers of functional decline, underscoring the importance of considering these relationships when using NfL for AD diagnosis or monitoring.